# Supplementary material for: Grip strength as a mediator in the relationship between physical activity and osteoporosis in older adults: Evidence from two longitudinal cohort studies
Source: PLoS One. 2026 Mar 24;21(3):e0340693. doi: 10.1371/journal.pone.0340693 (PMC13012474; doi:10.1371/journal.pone.0340693)
Supplement: S1 Table — a Activity type was selected based on examples showed to participants when investigating frequency of physical activity. b MET: metabolic equivalent of tasks. MET estimates were derived according to 2011 Compendium of Physical Activities. c Code represented exact type of activities, for activity corresponding to multiple potential types, Li et al. (2022) used mean of MET values from these activities. (DOCX) [file pone.0340693.s001.docx]

The physical activity (PA) scoring system proposed in this paper is an extension of Liu et al.'s (2022) methodology[^[[1]](#endnote-0)^].The researchers developed a physical activity (PA) scoring system consisting of three dimensions: First, they assigned a scoring standard for each intensity level (mild, moderate, or vigorous) based on different frequencies (1 = rarely/never, 2 = 1-3 times per month, 3 = weekly or more). Next, each score was weighted according to the average MET value for that intensity level (see Table S1 for details). The overall PA score was obtained by summing the scores from each dimension.[^[[2]](#endnote-1)^]

The ELSA used three questions to measure participation in mild, moderate, and vigorous physical activity, along with their frequencies. The answer options included: (1) rarely or never, (2) 1-3 times per month, (3) once a week, and (4) more than once a week. The HRS employed three questions related to physical activity, covering vigorous, moderate, or mild activity. The response options for these questions were: (1) daily, (2) several times per week, (3) once a week, (4) 1-3 times per month, and (5) never.

To harmonize the PA scoring between the two cohorts, we mapped the frequency of physical activity responses onto a unified 3-point scale (see S1 Table). For ELSA, responses were coded as follows: “rarely or never” = 1, “1–3 times per month” = 2, and “once a week or more than once a week” = 3. For HRS, responses were coded as: “never” = 1, “1–3 times per month” = 2, and “once a week or several times per week or daily” = 3.The scoring system is the same for all three activity categories. Second, standardized Z-scores are calculated by subtracting the respective mean value and dividing by the baseline standard deviation (SD) of the assigned scores. Third, to capture the differences between mild, moderate, and vigorous PA, the researchers summarized the weighted overall activity Z-scores. The weights were selected based on the estimated metabolic equivalent (MET) of the tasks[^[[3]](#endnote-2)^].After calculation, MET weights of 2.3, 4.4, and 7.2 were assigned to mild, moderate, and vigorous PA, respectively, which is consistent with previous studies. Detailed information on the weight calculation can be found in Supplementary Table S1.

# Table S1. Physical activity intensity categories, activity types, MET values, codes, and MET weights. Reproduced from Li et al. 2022.^1^

| Intensity | Activity type ^a^ | MET ^b^ | Code ^c^ | MET weight ^d^ |
| --- | --- | --- | --- | --- |
| Low | Laundry | 2·15 | Mean of 05090, and 05095 | 2·3 |
| Low | Home repairs | 2·50 | 06126 |  |
| Moderate | Gardening | 3·80 | 08245 | 4·4 |
| Moderate | Cleaning the car | 3·50 | 05020 |  |
| Moderate | Walking at a moderate pace | 5·30 | 17082 |  |
| Moderate | Dancing | 5·85 | Mean of 03010, 03014, 03019, 03020, 03025, 03030, 03031, 03038, 03040,03050, 03060 |  |
| Moderate | Floor or stretching exercises | 3·50 | 05130 |  |
| Vigorous | Running or jogging | 6·70 | Mean of 12010, 12020, 12025, 12027, 12150 | 7·2 |
| Vigorous | Swimming | 7·20 | Mean of 18230, 18240, 18310 |  |
| Vigorous | Cycling | 6·80 | 01011 |  |
| Vigorous | Aerobics or gym workout | 7·30 | 03015 |  |
| Vigorous | Tennis | 7·10 | Mean of 15675, 15680, 15690 |  |
| Vigorous | Digging with a spade or shovel | 7·80 | 08052 |  |

^a^ Activity type was selected based on examples showed to participants when investigating frequency of physical activity.

^b^ MET: metabolic equivalent of tasks. MET estimates were derived according to 2011 Compendium of Physical Activities.

^c^ Code represented exact type of activities, for activity corresponding to multiple potential types, Li et al. (2022) used mean of MET values from these activities.

1. [] Chenglong Li, Yanjun Ma, Rong Hua, Fanfan Zheng, Wuxiang Xie, Long-term physical activity participation trajectories were associated with subsequent cognitive decline, risk of dementia and all-cause mortality among adults aged ≥50 years: a population-based cohort study, Age and Ageing, Volume 51, Issue 3, March 2022, afac071, https://doi.org/10.1093/ageing/afac071 [↑](#endnote-ref-0)
2. [] Hamer, M., Terrera, G. M., & Demakakos, P. (2018). Physical activity and trajectories in cognitive function: English Longitudinal Study of Ageing. J Epidemiol Community Health, 72(6), 477-483. [↑](#endnote-ref-1)
3. [] Ainsworth, B. E., Haskell, W. L., Herrmann, S. D., Meckes, N., Bassett, D. R., Tudor-Locke, C., ... & Leon, A. S. (2011). 2011 Compendium of Physical Activities: a second update of codes and MET values. Med Sci Sports Exerc, 43(8), 1575-1581. [↑](#endnote-ref-2)
